# Sequence Instability in the Proviral Long Terminal Repeat and *gag* Regions from Peripheral Blood and Tissue-Derived Leukocytes of FIV-Infected Cats during the Late Asymptomatic Phase

**DOI:** 10.3390/vetsci3020010

**Published:** 2016-06-06

**Authors:** Christina D. Eckstrand, Chadwick Hillman, Brian G. Murphy

**Affiliations:** Department of Pathology, Microbiology, and Immunology, School of Veterinary Medicine, 4206 Vet Med 3A, University of California, Davis, CA 95616, USA; cnhillman@ucdavis.edu (C.H.); bmurphy@ucdavis.edu (B.G.M.)

**Keywords:** FIV, latency, promoter, LTR, *gag*, lymph node, asymptomatic, lentivirus

## Abstract

Feline immunodeficiency virus (FIV) infection results in viral persistence, a prolonged asymptomatic phase, and progressive immunopathology. During the asymptomatic phase, a cohort of experimentally FIV-infected cats exhibits features of viral latency in blood suggestive of inactive viral replication. We sought to investigate viral replication activity and genomic stability of the FIV proviral long terminal repeat (LTR) and the 5′ aspect of *gag* over time. FIV-infected cats during the asymptomatic phase demonstrated undetectable plasma FIV *gag* RNA transcripts and intermittent to undetectable blood-derived cell-associated FIV *gag* RNA. The LTR sequence demonstrated instability in blood-derived cells over time, in spite of low to undetectable viral replication. Sequence variation in the LTR was identified in CD4+ and CD21+ leukocytes from blood and surgically removed lymph nodes. Three single nucleotide polymorphisms (SNPs) in the LTR were commonly identified. Promoter functionality of a common LTR SNP and rare U3 mutation were examined by reporter gene assays and demonstrated either no change or increased basal FIV promoter function, respectively. In conclusion, this cohort of asymptomatic FIV-infected cats demonstrated instability of the LTR and 5’ *gag* sequences during the study period, in spite of undetectable plasma and rare to undetectable viral *gag* RNA, which suggests that blood may not accurately represent viral activity in asymptomatic FIV-infected cats.

## 1. Introduction

Feline immunodeficiency virus (FIV) of the family *Retroviridae*, subfamily *Orthoretrovirinae*, and genus *Lentivirus*, is the cause of a life-long, progressive immunocompromising disease affecting domestic cats worldwide [1,2]. FIV-infection typically progresses through three clinical phases in the cat; the acute, asymptomatic and terminal immunodeficiency phase [3]. In the acute phase of infection, there is prolific viral replication with broad viral distribution to lymphoid organs and many immune cell subsets including CD4+ T cells, CD8+ T cells, B-cells and monocytes/macrophages [3]. Clinically, the acute phase is characterized by high plasma viremia, a transient drop in peripheral blood CD4+ T cell numbers, fever and lymphadenopathy [3]. This is followed by the asymptomatic phase, which may last months to years, where the infected cat generally has no outward sign of clinical disease (clinical latency). However, on closer examination FIV-infected cats in the asymptomatic phase demonstrate multiple immunopathologies such as progressively declining numbers of peripheral blood CD4+ T cells, a persistently inverted CD4/CD8 ratio, cytokine aberrations and immune cell function abnormalities [3]. In the terminal immunodeficiency phase, viral replication rebounds in the face of immunopathology resulting in high plasma viremia, immune system failure, opportunistic infections or neoplasia [3]. The late asymptomatic phase of infection and associated viral activity remain under-investigated, possibly due to the cost of maintaining experimentally infected cats for prolonged periods of time. We sought to understand viral replication activity and the evolution of the proviral genome within infected cats during the asymptomatic phase. A more detailed understanding of this phase, along with that of the terminal immunodeficiency phase of disease, may facilitate the development of medical intervention strategies to prolong the asymptomatic phase or potentially eradicate the virus.

Our laboratory has established a cohort of experimentally FIV-infected cats where viral activity and immunopathology in the peripheral blood have been closely monitored over time. A detailed description of the acute phase and progression into the early asymptomatic phase for these cats has previously been reported on by our group [4]. In short, these cats reached a peak viremia approximately two weeks post inoculation that ranged from 4.9 × 104 to 5.8 × 106 copies vRNA/mL of plasma, followed by variable detection thereafter. Both proviral DNA and viral *gag* RNA were readily detectable in PBMCs. Approximately ten months post-inoculation, the cats entered the asymptomatic phase characterized by the absence of outward clinical disease and undetectable plasma viremia (clinical latency). We have identified several features of these infected cats that are consistent with the concept of viral latency including persistently undetectable plasma viral RNA, evidence that the FIV viral promoter is associated with a condensed chromatin pattern in peripheral blood CD4+ cells, and the presence of abundant short R-transcripts in CD4+ cells [3,4,5,6]. These features suggest that viral replication in the peripheral blood during the asymptomatic phase is either absent or present at a level below the limits of detection. It is a well-recognized and inherent feature of retroviral pathogenesis that proviral mutations accumulate as replication and cellular infection occur due to the lack of proofreading mechanisms in the viral polymerase, a relative infidelity of reverse transcription, and viral recombination *in vivo* [7,8,9]. As a result, it seems plausible that asymptomatic FIV-infected cats demonstrating persistently undetectable plasma viremia and viral latency in peripheral CD4+ cells would exhibit proviral sequence stability over time. We sought to further characterize viral activity in the peripheral blood during the asymptomatic phase by serially monitoring plasma and PBMC-associated viral *gag* RNA, and hypothesized that asymptomatic FIV-infected cats would demonstrate persistent inactive viral transcription in the peripheral blood, and genomic stability of the integrated provirus.

## 2. Materials and Methods

### 2.1. Animals and Sample Procurement

Six specific pathogen free cats were procured at approximately six months of age and housed at the University of California Davis Feline Research Colony facility. Four of the cats received an intramuscular injection containing 10^6^ TCID_50_ of FIV-C (Clade C Paddy-gammer strain) [4]. Two cats served as negative control animals and received an injection of virus-free sterile media. A previous report of cats experimentally infected with the FIV-C Paddy-gammer strain of virus indicated rapid disease progression, which did not occur in the cohort of cats reported here [10]. In fact, experimental infection with this strain resulted in quite the opposite. Our cats have experienced a very prolonged disease course with an extensive asymptomatic phase, which has given us the opportunity to investigate mechanisms of viral latency, tissue reservoirs, and viral evolution *in vivo*. Animals were monitored by physical examination for signs of illness, and peripheral blood collected biweekly to monthly. Approximately 10 months post-inoculation all of the cats entered the asymptomatic phase of infection where clinical signs of disease were absent (fever, cutaneous or gingival inflammation, lymphadenopathy, inappetence and/or lethargy) and plasma viral *gag* RNA was persistently undetectable by real-time PCR methods [4]. For this study, viral *gag* RNA transcription and proviral LTR sequence analysis were examined from PBMCs collected intermittently between the 150–300 weeks post-inoculation (wpi) study period. Between weeks 274–290 wpi, bilateral popliteal lymph nodes (PLNs) were surgically removed in order to compare lymphoid tissue-derived proviral LTR sequences to peripheral blood derived cells [11]. Proviral sequence analysis was extended to the 5′ aspect of the FIV *gag* segment (~1000 nucleotides) in lymph node-derived leukocytes, which was compared to the sequence of the inoculating virus. Based on our hypothesis, the expectation was that lymphoid tissue FIV proviral sequences (LTR and *gag*) would exhibit stability relative to the inoculating virus. All experimental study protocols were approved by the University of California Davis Institutional Animal Care and Use Committee (IACUC, permit #18155). The surgical pharmaceutical protocol included a subcutaneous premedication injection of atropine (0.02 mg/kg) and butorphanol (0.3 mg/kg), intravenous induction with ketamine (5 mg/kg) and midazolam 0.5 mg/kg, inhalational anesthesia maintenance with oxygen and 2% isoflurane via an endotracheal tube, and intraoperative intravenous ampicillin (20 mg/kg). Post-operative medications consisted of buprenorphine (0.02 mg/kg) administered transmucosally twice daily for 3–7 days as needed, and amoxicillin trihydrate/clavulanate potassium given orally twice daily (6.25 mg/lb) for 10 days.

### 2.2. Viral Transcription Status in Peripheral Blood over Time

At multiple time points between 150 and 300 wpi, 10 mL of blood were collected by jugular venipuncture and separated into plasma and PBMC fractions as previously described [4]. Viral RNA was extracted from 140 μL of plasma using a commercial kit (QIAamp Viral RNA Mini Kit, Qiagen, Valencia, CA, USA) and cell-associated RNA and DNA were co-extracted from 5 × 10^6^ PBMCs using the Allprep DNA/RNA Mini Kit (Qiagen). Plasma and PBMC fractions were examined for the presence of viral *gag* RNA by real-time polymerase chain reaction (PCR) methods [4], as the presence of detectable viral RNA in either fraction serves as evidence of active viral transcription. Between ten to fifteen plasma and eight to ten PBMC samples were collected from each FIV-infected and negative control cat during the study period with at least one month between collections for a particular cat. PBMCs from the FIV-infected cats were additionally interrogated between 258 and 262 wpi for the presence of short viral promoter proximal R-transcript RNA, a 66 nucleotide region of the LTR. Detectable short promoter proximal RNA transcripts, in the absence of detectable viral *gag* RNA, have been identified as a signature of viral latency as they indicate that RNA polymerase complex is paused on the promoter [5,12]. For this procedure, cell-associated RNA was extracted from 5 × 10^6^ PBMCs by the TRIzol method (Invitrogen, Carlsbad, CA, USA). All kit and TRIzol RNA extracts were DNAse treated (Turbo DNAse, Ambion, Carlsbad, CA, USA), reverse transcribed (Origene First Strand cDNA Synthesis Kit, Rockville, MD, USA) and interrogated by quantitative polymerase chain reaction (q-PCR) using FIV *gag*, feline GAPDH, and short R-transcript primers as previously described [4,5]. This real-time PCR assay has a detection limit of approximately 10 copies of FIV *gag* complimentary DNA (cDNA)/sample (data not shown) or 10^3^ copies of FIV *gag* cDNA per mL of feline plasma.

### 2.3. Sequence Instability of the Proviral LTR and gag Isolated from PBMC and PLN-Derived Leukocytes

The FIV LTR was PCR amplified from DNA extracted from PBMCs (as described above) and cloned using pCR2.1 TA cloning kit (Invitrogen) as previously described [6]. For each isolate, 5 clones were selected for sequencing. Amplified plasmid DNA was purified using a commercial kit (Wizard Plus SV Minipreps DNA Purification System, Promega, Madison, WI) and sequenced by a local vendor (Davis Sequencing, Davis, CA, USA). Sequences were analyzed for single nucleotide polymorphisms (SNPs), insertions, and deletions relative to the inoculating viral sequence.

In an attempt to further characterize the integrated provirus in the FIV-infected cats we examined the proviral LTR sequence of two known FIV cellular reservoirs, CD4+ and CD21+ leukocytes derived from both blood and popliteal lymph nodes at a single time point (between 274 and 290 wpi, depending on the cat). Surgical removal of the popliteal lymph node, leukocyte preparation and CD4+ and CD21+ leukocyte enrichment have been described previously [11]. DNA extraction, PCR amplification, cloning and sequencing procedures were as described above. Additionally, from unfractionated PLN-derived leukocytes, the first ~1000 nucleotides of the *gag* leader, *gag capsid* (CA) and 5′ terminus of *gag matrix* (MA) were PCR amplified, cloned and compared to the inoculating viral sequence using primers FIV _gag leader for_ (GTTGGCGCCCGAACAGGGA) and FIV _c rev_ (TAATGGGGATAGGGCTGACTCA).

### 2.4. Promoter Functionality Assays

We investigated the promoter functionality of the common _T_329_G_ SNP, and another unusual mutation detected from enriched blood-derived CD21+ leukocytes of cat 186 with a 52 base pair direct repeat insertion in U3 of the LTR. A beta-galactosidase reporter assay was performed to assess the functionality of the FIV promoter with these two individual mutations. From the plasmid shuttle vector described above (pCR 2.1, Life Technologies, Carlsbad, CA, USA) the FIV LTR clones were moved into a reporter plasmid (pBlue TOPO TA Expression Kit, Life Technologies). The reporter plasmid was used to assess the basal transcriptional activity of the inserted FIV promoters in transfected 293T cells (Lipofectamine 2000 Transfection Reagent, Life Technologies, Carlsbad, CA, USA). This was achieved by inserting the FIV promoter 5′ to the pBlue TOPO *lacZ* gene, which encodes a portion of the enzyme β-galactosidase. Expression experiments were performed in triplicate in 6-well tissue culture plates as described previously [13]. For each experiment, positive and negative control plasmids were transfected in parallel. The positive control used was referred to as CMV (pcDNA 3.1D/V5-His/lacZ, Life Technologies) containing a constitutively active cytomegalovirus promoter 5′ to the β-galactosidase reporter gene. The negative controls were either no plasmid (abbreviated “no tx”) or the β-galactosidase reporter plasmid lacking an upstream promoter (empty). Results for transfected wells were statistically evaluated by ANOVAs followed by Tukey multiple comparison tests to determine significant differences; a value of *p* < 0.05 was considered significant.

## 3. Results

### 3.1. Viral Transcription Status in Peripheral Blood over Time

**Figure 1:**
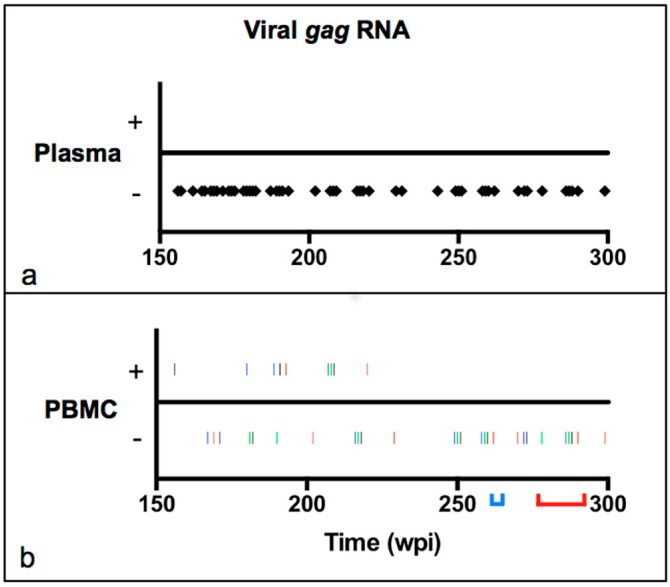
Plasma and PBMC-associated viral *gag* RNA in asymptomatic FIV-infected cats over time. (**a**) Viral *gag* RNA was undetectable by real-time PCR assays in the plasma of all four FIV-infected cats for the entire study period (150–300 weeks post-inoculation, each diamond represents a single plasma sample from an individual FIV-infected cat) and rarely, but intermittently detected in PBMCs between 150 and 220 wpi; (**b**) Viral *gag* RNA was intermittently detectable in the first half of the study period and undetectable in PBMCs between 220 and 300 wpi. Viral *gag* RNA was undetectable in both plasma and PBMCs during the periods that abundant short proximal promoter R-transcripts were amplified (blue bracket, 258–262 wpi) and the popliteal lymph nodes were surgically removed (red bracket; 274–290 wpi). Black = cat 187, blue = 184, red = 165, green = 186.

In order to examine the viral replication status in peripheral blood over time, approximately 10 mL of blood were collected by jugular venipuncture from four asymptomatic experimentally FIV-infected and two mock-infected negative control cats at multiple time points between 150 and 300 wpi and separated into plasma and PBMC fractions as described in the Materials and Methods. Plasma viral *gag* RNA was undetectable in all cats for the entire study period (150–300 wpi, Figure 1a). PBMC-associated viral *gag* RNA was detected in 9 out of 36 samples tested, and only within the first 70 weeks of the study period (between 150 and 220 wpi, Figure 1b). Each of the FIV-infected cats had at least one positive result, and at greatest three. PBMC-associated viral *gag* RNA was not detected in any sample between 220 and 300 wpi. Proviral *gag* DNA was detectable from PBMCs at all time points in FIV-infected cats. Neither viral *gag* DNA or RNA were detected at any time point in the plasma or PBMC samples from the uninfected negative control cats. Consistent with the concept of cellular latency, abundant short promoter-proximal R-transcripts were detected from unfractionated PBMCs of all four cats between 258 and 262 wpi, while FIV *gag* RNA remained undetectable (Table 1). Collectively these results indicate that there was a period between 150 and 220 wpi where viral transcription occurred intermittently but rarely within PBMCs, while viral RNA was undetectable in concurrently collected plasma samples. Collectively, these data are consistent with peripheral blood viral latency in the second half of the study period.

Abundant short promoter R-segment transcripts were amplified from all four FIV-infected cats, while viral *gag* transcripts were undetectable from peripheral blood PBMCs, consistent with viral latency and pausing of the RNA polymerase on the FIV promoter. Data are presented as the mean (±standard deviation) of triplicate PCR measurements per 10^6^ copies feline GAPDH cDNA, and are representative of PBMCs at 258–262 weeks post-infection for each cat. BLD indicates values below the limit of detection.

### 3.2. Sequence Stability of the Proviral LTR and gag Isolated from PBMC and PLN-Derived Leukocytes

**Figure 2:**
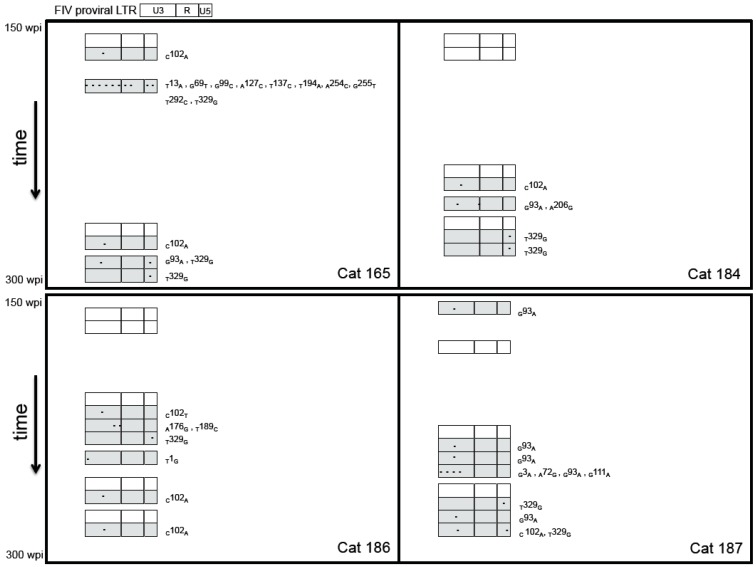
Serial analysis of amplified, cloned, and sequenced FIV proviral LTR segments from PBMCs of four FIV-infected cats over time during the asymptomatic phase (150–300 wpi). Each rectangle represents the FIV proviral LTR divided into the U3, R and U5 regions. Shaded rectangles indicate that the LTR sequence differed from the wild-type inoculating virus. Proviruses with point mutations were more commonly amplified than wild type (non-shaded rectangles). The approximate position of the SNP is denoted by an asterisk in U3, R, or U5 regions, with exact position and substituted base stated to the right of the shaded rectangle. Each rectangle represents a sequence derived from a single clone.

**Figure 3:**
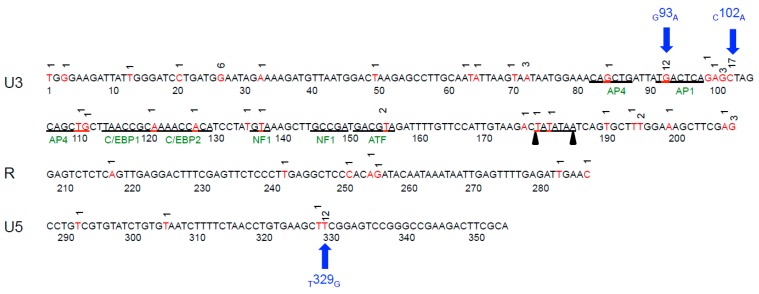
Summary of the FIV proviral LTR point mutations detected during the asymptomatic phase from all FIV-infected cats over time. Three SNPs were commonly detected (denoted by blue arrows). Nucleotide positions where mutations were detected are red and the number above the sequence indicates of number of times a particular mutation was detected. Known transcription factor binding sites are in green and the TATA box is underlined by black triangles.

To address the hypothesis that proviral genome stability would be maintained in asymptomatic FIV-infected cats over time, we PCR amplified, cloned and sequenced the entire FIV LTR from PBMC-derived DNA at multiple time points between 150 and 300 wpi. The proviral LTR sequence corresponding to the inoculating virus was rarely amplified from any of the infected cats throughout the study period (Figure 2). Proviral LTR sequences were examined for the presence of single/multiple nucleotide polymorphisms (SNPs), insertions and deletions. Single or multiple mutations were detected most frequently in the U3 region of the LTR, followed by the U5 and then the R segment. SNPs in the U3 region of the LTR occurred both within and outside of transcription factor binding sites (TFBS) and the TATA box. A summary of all SNPs detected in the FIV LTR from PBMCs is presented in Figure 3. Three SNPs were amplified (GenBank submission 1890989) most commonly including an adenine substitution for guanine at nucleotide 93 in U3 (G93A), occurring at an AP1 transcription factor-binding site; an adenine substitution for cytosine at nucleotide 102 (C102A); and a guanine substitution for thymine at nucleotide 329 of U5 (T329G). We have previously reported that the U5 SNP (T329G) was present as a rare variant in the inoculating virus stock of the cats [6], however it was not detected *in vivo* in any of the cats until after 150 wpi. Interestingly, the three most common SNPs were amplified from all four infected cats with the exception of the _G_93_A_ mutation, which was not amplified from cat 186.

**Figure 4:**
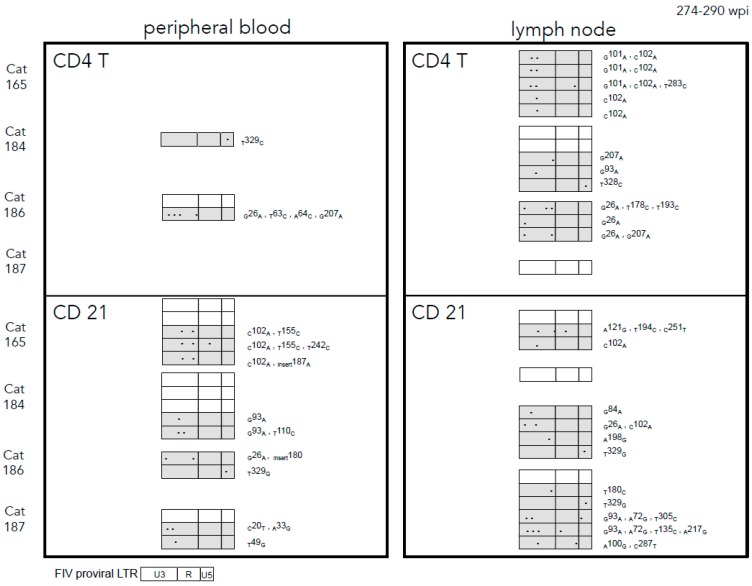
FIV proviral LTR variants in blood and popliteal lymph node-derived leukocyte subsets isolated from four asymptomatic FIV-infected cats (165, 184, 186, 187) between 274 and 290 wpi. Point mutations in the FIV LTR were detected in both leukocyte subsets of both compartments and most frequently occurred in the U3 region. Proviruses with point mutations or insertions were more commonly amplified than wild type (non-shaded rectangles). Shaded rectangles indicate that the proviral LTR sequence differed from the wild type inoculating virus. Proviral LTR DNA was not successfully amplified from blood-derived CD4+ cells in two of the FIV-infected cats (165, 187). Blood and popliteal lymph node tissues were collected concurrently from each cat. Each rectangle represents a sequence derived from a single clone.

**Figure 5:**
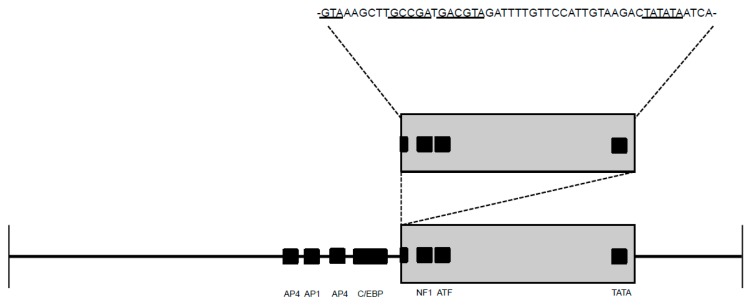
FIV proviral LTR mutated clone amplified from blood-derived CD21+ leukocytes from one cat (186). A 52 base pair direct repeat (grey rectangle) in U3 region includes both the NF1 (bipartite) and ATF transcription factor binding sites and TATA box (underlined and black boxes), resulting in duplication of these U3 sites.

When the proviral LTR sequences from enriched CD4+ and CD21+ leukocyte populations derived from blood or PLN were examined, we observed that mutated virus was more commonly amplified than wild-type virus from the infected cats’ leukocyte subsets with the exception of cat 184 CD21+ leukocytes. Again, the most frequently mutated region of the LTR was determined to be the U3 region in both cell types of both compartments (Figure 4). The three most commonly amplified LTR mutations (_G_93_A_, _C_102_A_, _T_329_G_) were amplified from both blood-derived and PLN leukocytes. The proviral LTR was difficult to amplify from peripheral blood CD4+ cells at the late 274–290 wpi time point; this was not the case for lymph node-derived CD4+ cells collected in parallel. This is likely due to the marked depletion of CD4+ cells in the peripheral blood over the course of infection (cat 165 had 98 CD4 cells/μL) and the rarity of FIV-infected peripheral CD4+ cells (estimated to be 1 infected cell in 1000 CD4+ cells) [5,6]. There were multiple SNPs unique to PLN-derived leukocytes, though mutations shared between blood and PLN leukocytes were just as commonly amplified. A unique and interesting 52 base pair direct repeat insertion in the U3 region resulting in a duplication of the NF-1 and ATF TFBS and the TATA box was amplified from PLN-derived CD21+ leukocytes from cat 186 (Figure 5).

**Figure 6:**
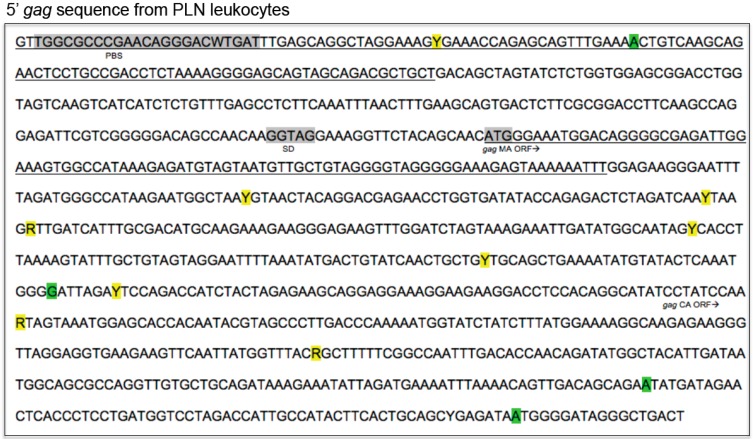
FIV 5′ aspect of the proviral *gag* consensus sequence derived from PLN leukocytes. The sequence is derived from unfractionated leukocytes from all four FIV-infected cats. Point mutations highlighted in yellow indicate SNPs and green indicates either a deletion or SNP was identified at this locus. “R” denotes nucleotide replacement with a purine base (A or G) and “Y” denotes replacement with a pyrimidine base (T or C). Primer binding site (PBS, grey box) and splice donor (SD, grey box) sites are represented. The start positions of the *gag MA* and *gag CA* open reading frames are denoted. Underlined sequences are required for efficient viral packaging [14].

The 5′ aspect of the FIV *gag* segment was PCR amplified (~1000 bp), cloned and sequenced from unfractionated PLN-derived leukocytes of all four cats. Multiple SNPs and deletions were identified relative to the inoculating virus (Figure 6, GenBank submission 1890989). Mutations were identified in regions known to be important for efficient encapsidation, and within the *matrix* and *capsid* open reading frames. Mutations did not appear to occur more commonly in any particular region.

### 3.3. Promoter Functionality Assays

**Figure 7:**
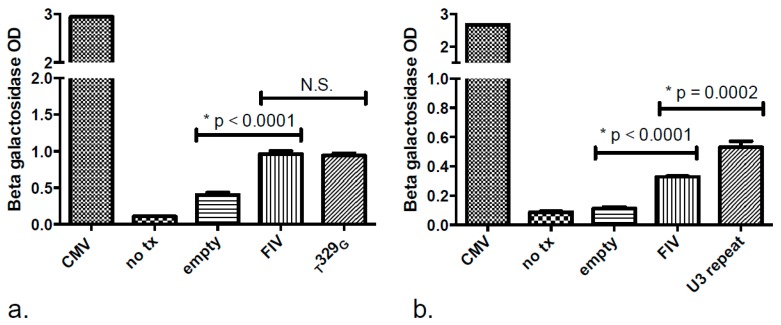
Point mutation _T_329_G_ results in no change, while the U3 insertional repeat in the FIV-C promoter results in augmentation of promoter function in an *in vitro* reporter assay. The promoter of the inoculating virus and the promoter with the _T_329_G_ SNP demonstrate strong basal promoter function in human 293T cells relative to negative controls (no transfection and a promoterless β-galactosidase plasmid-“no tx” and “empty”, respectively). The promoter function with the common _T_329_G_ SNP was not significantly (N.S.) different from the wild type inoculating FIV promoter (**a**); Cells transfected with the proviral U3 direct repeat mutant demonstrated a basal promoter function significantly above the wild type inoculating FIV promoter (**b**). Cells transfected with a CMV-β galactosidase plasmid (CMV) served as a positive control. An asterisk denotes statistical significance for selected pairwise comparisons (* *p* < 0.05) and error bars denote standard deviation.

Our group previously demonstrated that the _G_93_A_ LTR mutation within an AP-1 TFBS abolishes the basal promoter transcriptional activity, while the _C_102_A_ mutation has no apparent effect on the promoter relative to the wild type sequence [4]. We sought to similarly characterize the effect of the _T_329_G_ SNP and another unusual mutation detected from enriched blood-derived CD21+ leukocytes of cat 186 at 278 wpi, in which there was a 52 base pair direct repeat insertion in U3 of the LTR (Figure 5, GenBank submission 1890989). In transfected human fetal kidney cells (293T), the _T_329_G_ mutation had no effect on basal promoter expression levels relative to the wild-type FIV LTR, while the 52 base pair inserted repeat significantly amplified FIV promoter expression above basal promoter expression (Figure 7).

## 4. Discussion

In this study we demonstrated instability in the FIV proviral LTR and 5′ aspect of the *gag* region over time in the late asymptomatic phase, in spite of low cell-associated viral transcription in the peripheral blood in the first half of the study period and undetectable viral transcription in the second half. These findings are inconsistent with our hypothesis that asymptomatic FIV-infected cats would demonstrate persistent inactive viral transcription in the peripheral blood, and genomic stability of the integrated provirus. In fact, we demonstrated that these asymptomatic cats transitioned from a relatively quiescent but intermittently active viral replication status in the peripheral blood, to an inactive transcription status at around 220 wpi, and new mutations continued to arise in the proviral LTR sequence regardless of the viral activity status of the peripheral blood. This suggests either a level of ongoing viral replication below the level of detection in the peripheral blood, or alternatively, that ongoing viral replication occurs during the asymptomatic phase in solid tissue reservoir sites. Our research group has recently found evidence of the latter, that there is active viral replication occurring in the popliteal lymph node in the asymptomatic phase in spite of apparent peripheral blood latency [11]. However, we also recognize that due to the inherent limitations of the real-time PCR assay (1 × 10e3 copies/mL plasma) it is possible that replication is occurring in the peripheral blood below the level of detection of our assay. Previous investigations into the stability of the proviral LTR and *gag* regions by our group demonstrated minimal to no sequence variation between zero and 62 wpi [4]. These results are surprising, given that active viral replication was readily detectable in the peripheral blood (detectable plasma and cell-associated viral *gag* RNA) in the early infection stage. Perhaps proviral sequence variation was not detected in the early phase of infection because mutated variants were rare relative to a large proviral pool of the inoculating viral variant.

We repeatedly identified three SNPs within the proviral LTR in this cohort of experimentally infected cats. It is less surprising that the SNP within the U5 region (_T_329_G_) was commonly amplified, as it was discovered as a rare variant present in the inoculating virus stock, though it was not seen *in vivo* until the late asymptomatic phase of infection. Replication incompetent HIV proviruses have been shown to be the result of viral sequence changes such hypermutation or large sequence deletions [15]. Our research group has demonstrated that a SNP in a critical position (like a TFBS) can dramatically alter the transcriptional functionality of the promoter [4]. In the current study we identified an interesting naturally occurring insertional direct repeat within the U3 region that significantly augmented promoter functionality. This may be the result of duplication of specific TFBS, or alternatively, the presence of tandem TATA boxes within this mutated proviral clone. In other lentiviral systems, the presence or absence of certain TFBS within the proviral U3 region has been demonstrated to affect the ability of the viral promoter to respond to cytokine-mediated activation [13]. It is interesting that most of the LTR SNPs amplified from these cats occurred in the U3 region, which contains eight known TFBS. SNPs were detected in all TFBS. It was not determined if the amplified proviral sequences were transcriptionally (replication) competent. It would be interesting to know the effect on promoter functionality and replication competency of all of these variants.

It is also interesting to note that all three commonly amplified LTR SNPs were eventually detected in all four of our FIV-infected cats, with the exception of the _G_93_A_ mutation, which was not amplified from cat 186. The predisposing factor(s) that leads to the accumulation of these SNPs remains enigmatic, but it is unlikely to be dependent upon the individual animal. We have previously demonstrated that the mitogen concanavalin-A is able to induce the occurrence of the _G_93_A_ and _C_102_A_ proviral LTR mutations in *ex vivo* cultured CD4+ cells [6]. In the current study we also demonstrated that these proviral sequence mutations were present in multiple types of peripheral leukocytes (CD4+ and CD21+ cells) as well as in leukocytes derived from lymphoid tissues, consistent with previous literature which supports these leukocyte subsets as important viral reservoirs [11,16]. The significance of promoter SNPs identified solely within PLN-derived leukocytes is yet to be determined. One could postulate that they may alter or abrogate promoter functionality, or alternatively that the mutation may confer specific lymphoid tissue tropism. It was not surprising that proviral DNA was not amplified from peripheral blood at the 274–290 wpi time point from two FIV-infected cats because the absolute CD4+ count of cat 165 was extremely low (98 CD4+ cells/μL) and thus cell-associated provirus is very rare. The other cat (187) that we failed to amplify provirus from peripheral CD4+ cells has a very low total proviral load and is considered to be a long term non-progressor [11].

In addition to the many mutations amplified in the FIV LTR, we also detected multiple proviral mutations in the proximal ~1000 nucleotides of FIV *gag* from PLN-derived cells. Whether these *gag* mutations affected viral replication function was not determined. Our group previously documented proviral sequence stability of the same *gag* leader region during the early asymptomatic phase (up to 57 wpi) from PBMC-derived gDNA in this cohort of cats, similar to what was identified for the LTR [4]. The *gag* leader region contains coding segments required for efficient viral packaging, the primer binding site and splice donor sequences, but also includes a locus within the 3’ aspect of the FIV *gag* leader region not constrained by encapsidation sequence requirements and with no known gene coding function [14]. We did not detect any mutations in the primer binding or splice donor sites. Regions within the open reading frames of *MA* and *CA* can vary considerably (up to 84%) between different geographic isolates of FIV [17], which also suggests that strict genetic stability in the *gag* sequence is not essential for the virus. Tolerable laxity in the proviral *gag* sequence has been identified in other immunodeficiency inducing retroviruses. Queen *et al.* demonstrated that many *gag* mutations arising in chronically SIV-infected macaques remain replication competent [18]. As well, mutations predicted to reduce efficient packaging of HIV do not in fact do so [19].

## 5. Conclusions

In conclusion, this cohort of experimentally-FIV infected cats in the late asymptomatic phase demonstrates proviral genomic instability in the LTR and 5’ aspect of *gag* in spite of undetectable viral replication in the peripheral blood. Genomic mutations in the proviral LTR may affect the function of the FIV promoter and viral replication. However, some mutations may be silent. Collectively, these findings suggest that peripheral blood may not be an accurate indicator of viral activity during the asymptomatic phase. Further investigations in various tissue reservoirs and viral replication within tissues during the asymptomatic phase are warranted.

## Figures and Tables

**Table 1:** Real-time PCR amplifications of viral *gag* and short promoter R-segment cDNA isolated from PBMCs.

Cat #	PBMC *gag* cDNA	Short R Copies
165	BLD	8.4 × 10^5^ ± 3.6 × 10^5^
184	BLD	1.31 × 10^6^ ± 3.9 × 10^5^
186	BLD	1.69 × 10^7^ ± 4.8 × 10^6^
187	BLD	2.44 × 10^7^ ± 4.3 × 10^6^
